# Community treatment orders in the UK 5 years on: a repeat national survey of psychiatrists

**DOI:** 10.1192/pb.bp.115.050773

**Published:** 2016-06

**Authors:** Ritz DeRidder, Andrew Molodynski, Catherine Manning, Pearse McCusker, Jorun Rugkåsa

**Affiliations:** 1 Berkshire Healthcare NHS Foundation Trust, Reading, UK; 2 Oxford Health NHS Foundation Trust, Oxford, UK; 3 South London and Maudsley NHS Foundation Trust, London, UK; 4 Glasgow Caledonian University, Glasgow, UK; 5 Akershus University Hospital, Lørenskog, Norway

## Abstract

**Aims and method** Community treatment orders (CTOs) are increasingly embedded into UK practice and their use continues to rise. However, they remain highly controversial. We surveyed psychiatrists to establish their experiences and current opinions of using CTOs and to compare findings with our previous survey conducted in 2010.

**Results** The opinions of psychiatrists in the UK have not changed since 2010 in spite of recent evidence questioning the effectiveness of CTOs. Clinical factors (the need for engagement and treatment adherence, and the achievement of adherence and improved insight) remain the most important considerations in initiating and discharging a CTO.

**Clinical implications** Given the accumulating evidence from research and clinical practice that CTOs do not improve outcomes, it is concerning that psychiatrists' opinions have not altered in response, particularly given the implications for patient care.

Community treatment orders (CTOs) have been available in Scotland since 2005 and in England and Wales since 2008. The provision of powers to compel in the community was controversial within the healthcare and legal professions, among those who use services and among organisations working on their behalf. Indeed, an alliance was formed in 2008 in England and Wales of 32 different organisations from all these stakeholder groups to express concern regarding the proposed introduction.^1^ In Scotland a CTO can be commenced in the community, whereas in England and Wales it can only be made when a patient is discharged from involuntary treatment in hospital. The most common discretionary conditions written into the orders are to take medication and to see clinical team members, although powers are potentially wider.

In England and Wales the CTO is initiated by the clinician. This is entirely discretionary and there are no situations in which one must be used. In Scotland the decision to grant a CTO rests with the mental health tribunal, on the advice of clinicians and mental health officers. This allows considerable scope for variation in practice. Defferent factors influencing the variable use of CTOs have been proposed by Dawson,^2^ including:

- the legal structure of the CTO regime
- the community mental health services available
- the clinician's and practitioner's views about the possible impact of coercion on their relationships with patients
- the expectations of third parties.

Attitudes of psychiatrists in England and Wales were surveyed by Crawford *et al*^3^ in 2000, when 45% of the 1171 respondents were in favour of a system allowing CTOs. In our survey of psychiatrists in England and Wales shortly after the introduction of CTOs this proportion had risen to 60%.^4^

Since their introduction in England and Wales, CTOs have been used extensively. The Care Quality Commission reported that 18 942 CTOs had been used in England and Wales by 31 March 2013 and on that date 5218 patients were on a CTO.^5^ The overall number of patients subject to a CTO shows a year on year increase of 10%.^5^ The only randomised controlled study of the effectiveness of CTOs^1^ included 333 participants and demonstrated no difference between those subject to a CTO and those not over a 12-month period across a wide range of outcomes. The study has proved to be controversial and engendered a number of critical commentaries.^6^

In Scotland, where CTOs have been available for longer, their use has also increased, to the extent that in 2013–2014 they accounted for 41% of the total number of compulsory treatment orders, reflecting a significant drop in the number of hospital-based orders (the remaining 59% of all compulsory treatment orders) and consequently, a continued shift to the community for people subject to compulsion.^7^ Given these trends, we decided to compare and contrast the views of psychiatrists north and south of the border.

## Study design

We conducted a national survey using an adapted version of the instrument developed by Manning *et al*,^4^ which in turn was based on a survey of clinicians in New Zealand conducted 10 years after CTOs were introduced there.^8^ We aimed to determine psychiatrists' views regarding CTOs now and to assess any changes over time and differences across context.

## Method

### Sample

The link to the survey was sent by email (from the Royal College of Psychiatrists) to all consultant psychiatrists listed as members of the General Adult Psychiatry and the Rehabilitation and Social Psychiatry Faculties at the beginning of June 2014. A reminder was sent after 3 weeks. No responses were accepted after 8 weeks. To avoid any duplications only one response from any one IP address would be accepted.

### Survey instrument

We made minimal alterations to Manning's questionnaire to maximise comparability with that survey and with the earlier New Zealand study.^8^ The survey included questions asking the respondents to rate the importance of a range of factors influencing CTO practice or statements about CTOs using five-point Likert scales (from 1, very important/strongly agree to 5, not important at all/strongly disagree). Respondents were also asked about sociodemographic information and the number of CTOs they had applied for.

Statistical analysis was carried out using SPSS version 17 for Windows. A descriptive analysis was carried out with data presented as appropriate for the distribution (mean and standard deviation for normally distributed data, median and interquartile range for non-normally distributed data, and number and proportion for categorical data).

## Results

Of the 3534 psychiatrists emailed, 364 responses were received. The response rate (10.3%) is lower than that of the original survey (37%) and the New Zealand survey (44%), both of which used hard copy questionnaires and not electronic ones. Where appropriate, we compare our results with our first survey from 2010 and the New Zealand study.

The majority of the respondents (*n*=327) were based in England and Wales and 37 were from Scotland. These numbers approximately reflect the differing populations of the countries concerned. The ethnicity and gender of respondents did not differ significantly from the overall membership of the Royal College of Psychiatrists.^9,10^ Most (85%) had been working in psychiatry for longer than 10 years.

The mean number of CTOs applied for was 15, with a reported range from 0 to 100. There was significant variation between posts in terms of the use of CTOs in routine practice, with understandably marked differences between, for example, in-patient and community services. The overwhelming majority of respondents (86%) reported that none of their applications for CTOs had been turned down.

Many respondents added written comments, suggesting high levels of interest in the topic, as also reported in our previous survey.

### CTO impact on the therapeutic relationship

When asked about the therapeutic relationship, 24% of respondents in England and Wales believed that CTOs help the relationship, 10% that they hinder it and 56% that they both help and hinder it. In Scotland the figures were 18%, 0% and 76% respectively.

### Important factors in the decision to use a CTO

The survey presented 11 factors and asked the respondent to rate their relative importance when deciding to use a CTO. The most important factor for responders in England and Wales was ‘To ensure contact with mental health professionals’, and ‘Promoting compliance with medication’ was ranked second. The five reasons deemed most important were the same in all four surveys over time and location. These results are summarised in Table 1.

**Table 1:** The mean rating (and listed rank) of importance attached by psychiatrists to key factors in decision to use a community treatment order^a^

	Scotland 2014	England and Wales 2014	England and Wales 2010	New Zealand 2002^b^
To ensure contact with mental health professionals	1.25 (2)	1.7 (1)	1.86 (3)	1.79 (1)

To promote adherence to medication	1.19 (1)	1.71 (2)	1.78 (1)	2.03 (4)

To protect from consequence of relapse	1.47 (4)	1.74 (3)	1.83 (2)	2.08 (5)

To ensure rapid detection of relapse	1.84 (5)	2.10 (4)	2.24 (5)	1.90 (3)

To provide the authority to treat the patient	1.31 (3)	2.27 (5)	2.08 (4)	1,81 (2)

To facilitate readmission to in-patient care	2.75 (10)	2.41 (6)	2.56 (6)	2.43 (7)

To reduce the risk of violence to others	2.56 (6)	2.42 (7)	2.61 (7)	2.68 (8)

To provide greater security for patients' families and caregivers	2.56 (6)	2.56 (8)	2.70 (8)	2.41 (6)

To reduce the risk of self-harm by the patient	2.56 (6)	2.6 (9)	2.81 (9)	2.74 (9)

To enhance the obligation of service providers to the patient	2.63 (9)	2.97 (10)	3.12 (10)	2.97 (10)

To help ensure police assistance with patients will be available	4.03 (11)	3.72 (11)	3.74 (11)	3.31 (11)

a. Rating: 1, very important; 3, neutral; 5, not important.

b. 10 years post-introduction.

Thirty-four respondents added additional reasons such as ‘to make the patient feel more secure’, ‘to establish insight’ or ‘to gradually promote a sense of independence’.

### Important factors in the decision to discharge a patient from a CTO

Development of insight and adherence to treatment were rated as the most important factors in discharging patients from CTOs. The mean ratings suggest that these were considered very important. Again, the five most important factors were the same in all four surveys. These results are summarised in Table 2.

**Table 2:** The mean rating (and listed rank) of the importance attached to factors in the decision to discharge a community treatment order^a^

	Scotland 2014	England and Wales 2014	England and Wales 2010	New Zealand 2002^b^
Development of insight	1.17 (1)	1.53 (1)	1.61 (1)	1.56 (2)

Adherence to treatment	1.17 (1)	1.60 (2)	1.61 (1)	1.53 (1)

Clinical improvement	1.33 (3)	1.60 (2)	1.61 (1)	1.58 (3)

Reduced risk to others	1.83 (4)	1.88 (4)	2.04 (4)	1.84 (4)

Reduced risk to self	1.97 (5)	1.98 (5)	2.12 (5)	1.87 (5)

Suitable accommodation and community supervision	2.07 (6)	2.05 (6)	2.18 (6)	2.12 (6)

Reduced substance use	2.07 (6)	2.06 (7)	2.31 (7)	2.25 (7)

Improved lifestyle	2.33 (8)	2.12 (8)	2.41 (8)	2.65 (11)

To increase the patient's freedom	2.60 (11)	2.29 (9)	2.53 (10)	2.84 (12)

Employment	2.67 (12)	2.37 (10)	2.41 (8)	2.60 (9)

Improved family relationships	2.33 (8)	2.41 (11)	2.64 (11)	2.32 (8)

Enhanced social/cultural networks	2.47 (10)	2.47 (12)	2.71 (12)	2.64 (10)

The patient's desire to be discharged	2.93 (13)	2.49 (13)	2.81 (13)	3.09 (14)

Suitable recreational activities (including exercise)	2.93 (13)	2.80 (14)	3.14(14)	3.02 (13)

Enhanced cultural identity	3.47 (15)	2.97 (15)	3.42 (15)	3.19 (15)

a. Rating: 1, very important; 3, neutral; 5, not important.

b. 10 years post-introduction.

### Mechanisms influencing how CTOs work

Respondents were presented with nine possible mechanisms by which CTOs may affect outcome. The three mechanisms deemed most important were ensuring medication adherence, ensuring a greater period of stability, and signalling to the patient that they have severe mental illness. In Scotland the first two were endorsed but the third most influential factor was the binding of services to the patient, with ‘signalling’ being just behind in fourth position. These results are summarised in Table 3.

**Table 3:** The mean rating (and listed rank) of the importance attached to factors influencing how community treatment orders work^a^

	Scotland 2014	England and Wales 2014	England and Wales 2010
Ensures medication adherence for a lengthy period during which other changes can occur	1.3 (1)	1.90 (1)	1.95 (1)

Ensures a greater period of stability	1.57 (2)	2.12 (2)	2.26 (2)

Signals to patient that they have a SMI which needs active management	2.40 (4)	2.27 (3)	2.28 (3)

Commits service providers to the patient	2.40 (4)	2.66 (4)	2.76 (4)

Gives others the confidence to care for the patient	2.63 (7)	2.75 (5)	2.79 (5)

Binds community mental health services into place	2.37 (3)	2.80 (6)	2.89 (6)

Mobilises social support for the patient	2.60 (6)	2.97 (7)	3.25 (8)

The patient gives up key conflict areas to external agents	3.07 (8)	3.07 (8)	3.30 (9)

Encourages the patient to take responsibility	3.30 (9)	3.08 (9)	3.15 (7)

SMI, serious mental illness.

a. Rating: 1, very important; 3, neutral; 5, not important.

### The importance of factors that may undermine the effectiveness of CTOs

The results here are the same as our earlier survey, with substance misuse (mean rating 1.92), lack of adequate supported accommodation (mean rating 2.23), and failure to enforce adherence (mean rating 2.31) being seen as most important. The ranking of factors was the same in both England and Wales surveys and in the Scottish 2014 sample.

### Factors important in the introduction of CTOs

The rank order of perceived significance of factors behind the introduction of CTOs as listed in this survey was as follows for respondents in England and Wales (a rating of 1 indicates strong agreement, 3 indicates a neutral stance and 5 strong disagreement; mean ratings are given in brackets: the lower the mean rating, the higher the factor significance):

1. to enforce better community services and follow-up for those most at risk (2.22)
2. as a response to public pressure (in respect to acts of violence committed by mentally ill patients) (2.45)
3. as a result of procedural evolution – moving from Sections 17 and 25 (of the Mental Health Act 1983) to more explicit framework for treating those with mental illness in the community (2.51)
4. to reduce pressure on acute psychiatric beds (2.89)
5. to reduce coercion by reducing length of stay in psychiatric hospitals (2.95)
6. as a result of international research evidence (3.53).

There is no difference in the listed ranking from our first survey.^4^ The results for Scottish respondents are different in that the ‘public pressure’ factor is rated of less importance (3.31).

#### Support for the introduction and use of CTOs

We asked clinicians to rate their level of agreement with several statements regarding CTOs (mean ratings are given in brackets):

1. ‘CTOs complement existing legislation to provide a greater choice of treatment options’ (2.37)
2. ‘The increase in compulsory powers is appropriate for the potential clinical and social benefits’ (2.40)
3. ‘I supported the introduction of CTOs’ (2.55)
4. ‘There is sufficient clinical guidance to feel confident placing patients on CTO’ (2.60)
5. ‘I can already see the benefits of the use of CTOs’ (2.63).

Those statements with a mean of over 3, suggesting disagreement, were:

1. ‘The introduction of CTOs will have no long-term benefit’ (3.17)
2. ‘Well-resourced community services can provide the same benefits without using compulsory powers’ (3.22)
3. ‘There is insufficient clinical evidence to feel comfortable placing patients on CTO’ (3.46)
4. ‘The introduction of CTOs was a retrograde step for mental health services’ (3.59).

### Factors discouraging clinicians from using CTOs

Mean scores here tended to cluster around the mid-range, suggesting less clearly held beliefs and opinions than in other areas of the survey. The most important discouraging factors were considered to be concern for civil liberties (mean rating 2.29), administrative burden (mean rating 2.31), and the degree of coercion involved (mean rating 2.41). Lack of evidence to support effectiveness was ranked fourth most important (mean rating 2.51). No other factors had mean ratings below 3 (a level that suggests general agreement). Comparing the four surveys across time and geography, there are again remarkably few differences.

### Should Section 17 or CTOs be used in community care?

Respondents in England and Wales were asked to compare the use of CTO and Section 17 leave from hospital: 58% believed that CTOs were a more appropriate way of treating patients in the community than the medium- to long-term use of Section 17 leave and the majority believed they were less coercive (48% *v*. 23%). There was widespread belief (expressed by 61% of respondents) that in clinical care there was a significant difference in powers between Section 17 leave and CTOs.

### Contrasts between jurisdictions

There was some variation in opinions expressed on CTOs incorporating medical treatment that is ‘likely to prevent a disorder worsening or likely to alleviate the symptoms or effects of the disorder’: 47% of respondents from England and Wales and 68% of Scottish respondents agreed. Further, 84% of Scottish respondents agreed that it should be possible to place a patient not currently under detention on a CTO, but only 36% of respondents in England and Wales did so. There were also differences between jurisdictions regarding significantly impaired decision-making ability (SIDMA) as a criterion for CTO use: 80% of psychiatrists in Scotland and 28% of those in England and Wales accepted it. This is a criterion in the Scottish legislation (Mental Health (Care and Treatment) (Scotland) Act 2003) but not in England and Wales (Mental Health Act 1983). Even though the number of respondents from Scotland was relatively small at 37, and we did not believe as a result that detailed analysis was appropriate, important differences do appear to be present in attitudes and beliefs.

## Discussion

The results of this survey are strikingly similar overall to the previous survey in England and Wales conducted when these powers were relatively new in those countries. Nearly 6 years on, with no measurable reductions in bed use or improved outcomes from clinical practice or research,^11–15^ the use of CTOs continues to increase.^5,6^

It is likely that the reasons for this are complex and relate to factors within both clinicians and the systems they operate in. In a discretionary system where there is never a situation where a CTO must be enacted, the opinions and beliefs of senior clinicians, particularly psychiatrists, are likely to be influential. Clinical factors remain by far the most important in decisions to initiate or discharge a CTO. These have been the same in all four studies spanning time and geography.^3,4,8,16^ The key factors were as follows: to ensure contact with professionals, to promote medication adherence, and to identify relapse and protect the individual from its consequences. Similarly, the factors in deciding when to discharge a CTO have been remarkably stable: development of insight, treatment adherence, clinical improvement and reduced risk to others and self (in that order).

Clinicians reported feeling discouraged by concerns for civil liberties and the additional administrative burden when considering CTOs. Factors believed to undermine CTOs were substance misuse, lack of supported accommodation and failure to enforce adherence.

The written comments of a number of respondents expressed frustration around how the in-patient/out-patient split complicates practice. A number commented also that a general pressure on beds may lead to inappropriate use of CTOs to facilitate early discharge or to make readmission ‘easier’ if needed. Clearly these raise ethical concerns if compulsory powers are being commonly used as a practical measure to access services that should be available regardless of legal status. It has also been reported anecdotally that clinicians feel pressurised to use CTOs in case there is a negative outcome such as suicide or aggression and they are criticised for not having done so.

It seems likely that clinicians across the UK will continue to use CTOs in large numbers as long as they remain available. New evidence, both in the form of large-scale utilisation data and research studies,^1,7,17^ has not affected opinion significantly. This is of concern in a branch of medical science that ostensibly embraces evidence-based practice. It seems crucial therefore that we continue to evaluate our practice,^18^ gather good-quality evidence, and pay attention to it to facilitate greater understanding and sophistication in the use of compulsion. This will better serve our patients.
